# SpecGMM: Integrating Spectral analysis and Gaussian Mixture Models for taxonomic classification and identification of discriminative DNA regions

**DOI:** 10.1093/bioadv/vbae171

**Published:** 2024-11-05

**Authors:** Saish Jaiswal, Hema A Murthy, Manikandan Narayanan

**Affiliations:** Department of Computer Science and Engineering, Indian Institute of Technology (IIT) Madras, Chennai 600036, India; Department of Computer Science and Engineering, Indian Institute of Technology (IIT) Madras, Chennai 600036, India; Department of Computer Science and Engineering, Shiv Nadar University, Chennai 603110, India; Department of Computer Science and Engineering, Indian Institute of Technology (IIT) Madras, Chennai 600036, India; Center for Integrative Biology and Systems Medicine, IIT Madras, Chennai 600036, India; Robert Bosch Centre for Data Science and Artificial Intelligence, IIT Madras, Chennai 600036, India

## Abstract

**Motivation:**

Genomic signal processing (GSP), which transforms biomolecular sequences into discrete signals for spectral analysis, has provided valuable insights into DNA sequence, structure, and evolution. However, challenges persist with spectral representations of variable-length sequences for tasks like species classification and in interpreting these spectra to identify discriminative DNA regions.

**Results:**

We introduce SpecGMM, a novel framework that integrates sliding window-based Spectral analysis with a Gaussian Mixture Model to transform variable-length DNA sequences into fixed-dimensional spectral representations for taxonomic classification. SpecGMM’s hyperparameters were selected using a dataset of plant sequences, and applied unchanged across diverse datasets, including mitochondrial DNA, viral and bacterial genome, and 16S rRNA sequences. Across these datasets, SpecGMM outperformed a baseline method, with 9.45% average and 35.55% maximum improvement in test accuracies for a Linear Discriminant classifier. Regarding interpretability, SpecGMM revealed discriminative hypervariable regions in 16S rRNA sequences—particularly V3/V4 for discriminating higher taxa and V2/V3 for lower taxa—corroborating their known classification relevance. SpecGMM’s spectrogram video analysis helped visualize species-specific DNA signatures. SpecGMM thus provides a robust and interpretable method for spectral DNA analysis, opening new avenues in GSP research.

**Availability and implementation:**

SpecGMM’s source code is available at https://github.com/BIRDSgroup/SpecGMM.

## 1 Introduction

Understanding the information encoded in genomic sequences to elucidate their structure, function, and evolution is a fundamental goal of bioinformatics (Durbin *et al.* 1998). A variety of computational techniques, including string comparison algorithms, probabilistic models like hidden Markov models, signal processing methods, and machine/deep learning approaches, have been employed toward this end. Recent advances in deep learning have inspired large language models that generate numerical vector representations for biomolecular sequences, which can then be used for various downstream tasks (Alharbi and Rashid 2022, Yue *et al.* 2023), including species classification. But these approaches often lack transparency in their decision-making processes, despite advances in interpretable deep learning models (Novakovsky *et al.* 2023). On the other hand, Genomic signal processing (GSP) offers a unique perspective by viewing DNA/RNA/protein sequences as signals and representing them in the frequency/spectral domain (Anastassiou 2001, Vaidyanathan 2004). GSP methods help reveal informative features and repetitive patterns in sequences, lending themselves well to interpretability, a crucial aspect in understanding biological functions and discriminative features across species for classification tasks. GSP has proven particularly useful for tasks such as gene identification (Vaidyanathan 2004), structure analysis (Zhang *et al.* 2002), and, importantly, taxonomic classification (Randhawa *et al.* 2019, 2020, Kar and Ganguly 2024)—the focus of this study.

Despite their advantages, existing GSP-based methods face several challenges in the context of taxonomic classification which include (i) limited diversity in datasets analyzed that can reduce generalizability on diverse datasets, (ii) issues with handling sequences of greatly varying lengths without losing sequence information, and (iii) challenges in identifying and interpreting discriminative DNA features learnt by the classification models. For instance, Skutkova *et al.* (2013) utilized Dynamic Time Warping to classify genomic signals corresponding to *ACTA1* gene across a dataset of 10 organisms, mostly mammals. Their approach showcased promising results, but is limited by its focus on a single gene and a small set of species. On the other hand, Randhawa *et al.*’s GSP studies (Randhawa *et al.* 2019, 2020) on a larger set of species accommodate sequences of vastly differing lengths through length normalization techniques like truncation or padding. But these techniques often lead to loss or distortion of sequence information. Further, Randhawa *et al.* do not adequately address the interpretability of spectral features used for classification. Earlier works, such as those by Sussillo *et al.* (2004) and Hassani Saadi *et al.* (2017), have used spectrograms to uncover and interpret unique visual patterns and characteristic features of DNA sequences. Yet, these studies primarily analyze spectrograms of individual sequences or specific regions, without systematically comparing spectrograms from sequences of different organisms. Thus, there is a clear need for a systematic approach that compares and contrasts spectral features of sequences from different organisms to enhance interpretability and applicability of GSP-based taxonomic classifiers.

We introduce SpecGMM, a novel framework that integrates GSP with Gaussian Mixture Models (GMM) to classify DNA sequences across various taxonomic levels/ranks. By using a sliding window technique and a background GMM, SpecGMM preserves most of the sequence information when converting variable-length DNA sequences to fixed-dimensional spectral representations. The resulting spectral features are also amenable to comparative spectrogram analysis and visualization. Our evaluations using diverse DNA sequence datasets demonstrate the superior performance of SpecGMM over a baseline GSP method in most cases, thereby providing not only improved accuracy (of up to 35.55% for SpecGMM’s Linear Discriminant (LD) classifier) but also deeper insights into the discriminative patterns within DNA sequences. Our approach thus promises to broaden the applicability of GSP in genomic research, paving the way for new research in taxonomic classification and beyond.

## 2 Methods

This section describes our SpecGMM framework, which uses GSP integrated with GMMs to effectively obtain spectral representation for DNA sequences. Application of sliding window technique on variable-length sequences preserves most of the sequence information for robust classification. We assess SpecGMM’s performance across diverse datasets, comparing it with a baseline method, and focus on analyzing spectral features to interpret the biological significance of the results.

### 2.1 Background on genomic signal representation and baseline classification techniques

Genomic (DNA) sequences are transformed into discrete signals using numerical representations (see the first table in Randhawa *et al.* 2019), thus enabling the exploitation of signal processing techniques. This study utilizes the Purine-Pyrimidine (PP) representation, where purines (A, G) are assigned a value of −1 and pyrimidines (T, C) a value of 1 (see Supplementary Fig. S1A). Nucleotides marked as unknown (N) are excluded from the analysis. This representation allows for the application of digital signal processing techniques, specifically through the use of the Fast Fourier Transform (FFT) algorithm to efficiently compute the Discrete Fourier Transform (DFT) and analyze the spectral characteristics of these signals. Please refer to Supplementary Methods: Sections 1.1 and 1.2 for further details on the application of DFT to derive magnitude spectrum.

The baseline method involves normalizing the length of sequences to median length of all sequences either by truncation or anti-symmetric padding (see Supplementary Fig. S1B) to facilitate the extraction of fixed-dimensional magnitude spectra. The baseline method constructs a pairwise distance matrix using magnitude spectra for training sequences for feature extraction. Columns of the distance matrix are used as features to train six different machine learning classifiers—Fine K-Nearest Neighbor (KNN), Subspace KNN, LD, Subspace Discriminant, and two Support Vector Machine (SVM) models, viz., Linear SVM (LSVM) and Quadratic SVM (QSVM). However, this method could result in the loss of significant information for sequences longer than the median. Additionally, increasing the number of sequences in the training set complicates the classification task due to the expanding dimensionality of distance matrix-based feature vectors.

### 2.2 SpecGMM method

**Figure 1.**
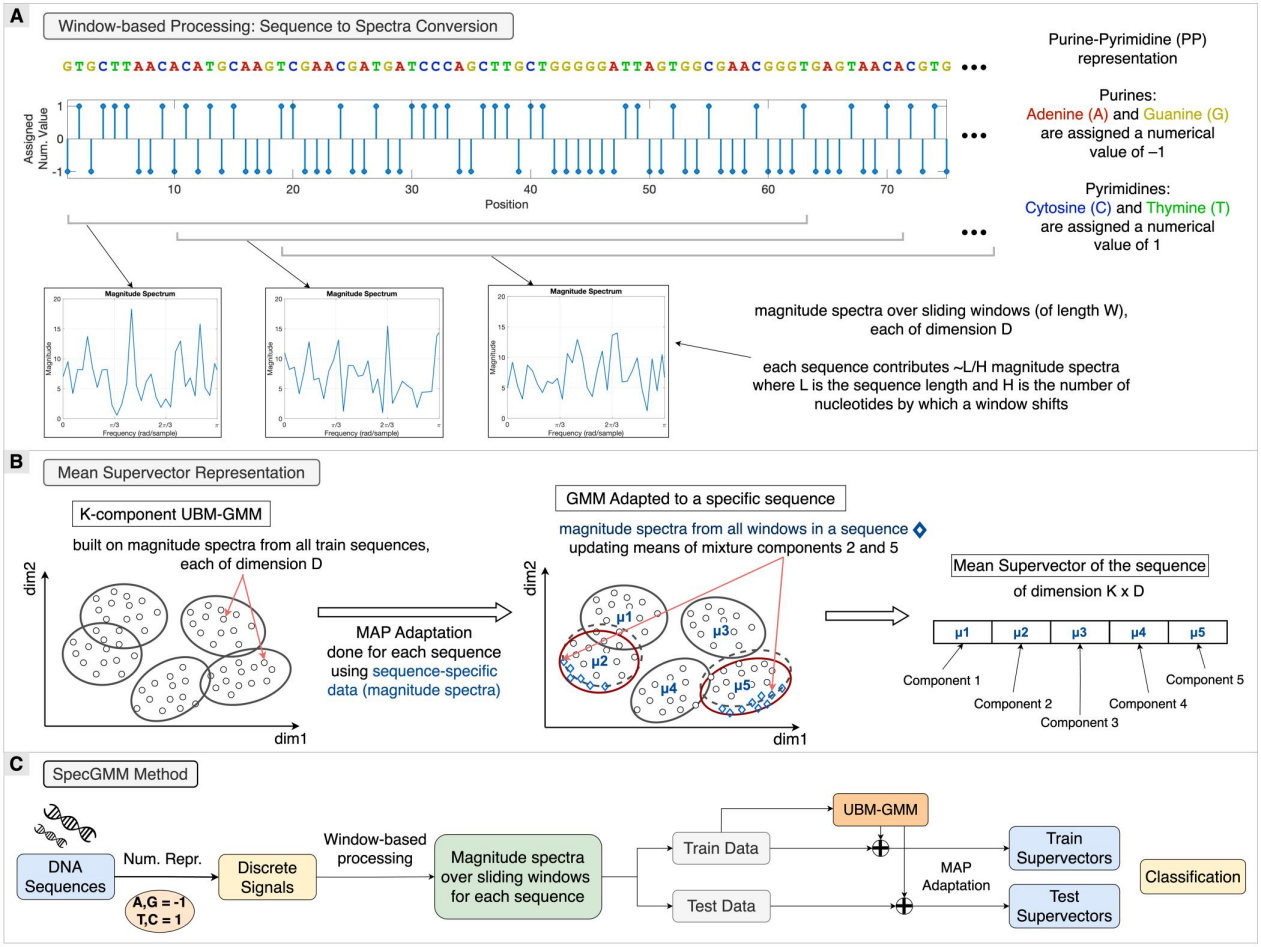
Overview of the SpecGMM methodology: (A) PP representation is used to transform the DNA sequence into a discrete signal, as discussed in Section 2.1. A fixed-size window slides across sequence length, and DFT is applied to extract magnitude spectra at each position, revealing local frequency content. (B) A background GMM (termed UBM-GMM for Universal Background Model—GMM) is constructed from class-wide spectra, with individual sequence GMMs adapted via MAP adaptation to produce mean supervectors. (C) The classification workflow involves converting DNA sequences into signals, extracting spectra via window-based processing, building a UBM-GMM with training data, adapting it to obtain mean supervectors for training and testing, and employing these supervectors to train classifiers and assess their performance on test data. The method is detailed in Section 2.2.

In contrast to the baseline approach, our proposed SpecGMM framework enhances species classification by leveraging a Universal Background Model (UBM)-GMM to process sliding window-based spectral features from DNA sequences of varying lengths and project them into a fixed-dimensional feature space (Fig. 1). This approach preserves most of the sequence information and addresses the issue of sequence length normalization. Moreover, it effectively manages the dimensionality of sequence-specific feature representations without depending on the number of training sequences, showcasing a significant advancement over the baseline classification method.

#### 2.2.1 Computing spectral features using sliding windows

SpecGMM begins by analyzing DNA sequences through a systematic application of fixed-length sliding windows, capturing spectral data across the entire sequence. This method moves a fixed-length window, which encompasses a predetermined number of DNA nucleotides, across the sequence, allowing overlaps to ensure continuous feature coverage. For each window, the magnitude spectrum is computed with the FFT order adjusted to the nearest power of two, focusing on the first half of the spectrum (0 to *π*) due to its symmetry (Supplementary Fig. S1A). This process generates a sequence of fixed-dimensional (*D*-dimensional) spectra for each DNA sequence, as shown in Fig. 1A. While averaging these spectra over all windows may provide a sequence-specific fixed-dimensional representation, it may obscure local spectral features. To preserve these local features when reducing dimensionality, we employ a *K*-component UBM-GMM framework to obtain a better fixed-dimensional ((K×D)-dimensional) feature representation for each sequence.

#### 2.2.2 Fixed-dimensional representation using UBM-GMM

A UBM-GMM is a GMM extensively used in speaker recognition to distinguish speakers by creating fixed-dimensional feature representations from variable-length speech utterances, as described by Reynolds *et al.* (2000). Inspired by this property, we apply UBM-GMM to genomic data, enabling fixed-length representations of variable-length DNA sequences. Constructing a GMM requires extensive data for accurate parameter estimation, especially for high-dimensional feature vectors. To address this issue, UBM-GMM is constructed with the Expectation-Maximization algorithm (detailed in Supplementary Algorithm 1), by aggregating data across multiple classes to ensure parameter robustness. Features from a specific DNA sequence are then given as input to this model through Maximum a Posteriori (MAP) adaptation (Supplementary Algorithm 2), adjusting only the means of the GMM mixture components to create a mean supervector for each sequence (Fig. 1B). This mean supervector represents the fixed-length feature vector for the sequence, eliminating the need for sequence truncation or padding.

#### 2.2.3 Classification using mean supervector representation

The mean supervector feature representations for sequences obtained through SpecGMM are used for the classification tasks (see Fig. 1C). This method ensures that the feature vector dimension does not depend on the number of training sequences. This addresses a significant limitation of the baseline approach. To ensure direct comparisons with the baseline method, we use the classifiers used in the baseline study. SpecGMM’s time-complexity analysis and hyperparameter selection process are explained in Supplementary Methods: Sections 1.5 and 1.6.

### 2.3 Datasets

Our SpecGMM framework was tested on diverse datasets, including mitochondrial DNA (mtDNA) sequences, viral and bacterial genomes, and 16S ribosomal RNA (rRNA) sequences. All datasets except 16S rRNA sequences were obtained from Randhawa et al.’s studies (Randhawa *et al.* 2019, 2020). The bacterial 16S rRNA sequences were obtained from the 16S-ITGDB dataset (Hsieh *et al.* 2022). These 16S rRNA sequences were preprocessed to identify hypervariable regions (HVRs) and retain only sequences containing HVRs V2–V7 for further analysis. We used QIIME2 toolkit (Bolyen *et al.* 2019) with the primer information from Chaudhary *et al.* (2015) to identify HVRs V2–V7, and excluded analysis of regions V1, V8, and V9 due to inconsistencies in the available primers. More details of the preprocessing are in Supplementary Methods: Section 1.7 and Supplementary Table S1. Descriptive statistics for datasets are provided in Supplementary File D1. Additional information on the 16S rRNA sequences, such as taxonomy labels and start/end positions of the HVRs obtained using QIIME2, is in Supplementary File D2.

### 2.4 SpecGMM evaluation strategy

A stratified four-fold cross-validation method was used to optimize SpecGMM’s hyperparameters using only a dataset of plant mtDNA sequences. This Plants dataset comprises two categories, Chlorophyta with 44 sequences and Streptophyta with 130 sequences, and was chosen for hyperparameter tuning due to the high variability of its sequences. To prevent data leakage, we froze the hyperparameters obtained from the Plants dataset and applied them to other genomic datasets. Further, these datasets were also divided into four stratified folds, with three folds used for training and one for testing. This approach ensured that each fold served as an independent test set once. We computed performance metrics such as accuracy, precision, recall, specificity, and F1-score for each test scenario.

## 3 Results

We performed extensive analysis across various genomic datasets to assess the efficacy of the SpecGMM approach for DNA sequence-based classification tasks.

### 3.1 Hyperparameter optimization and illustrative performance for plant species classification

**Figure 2.**
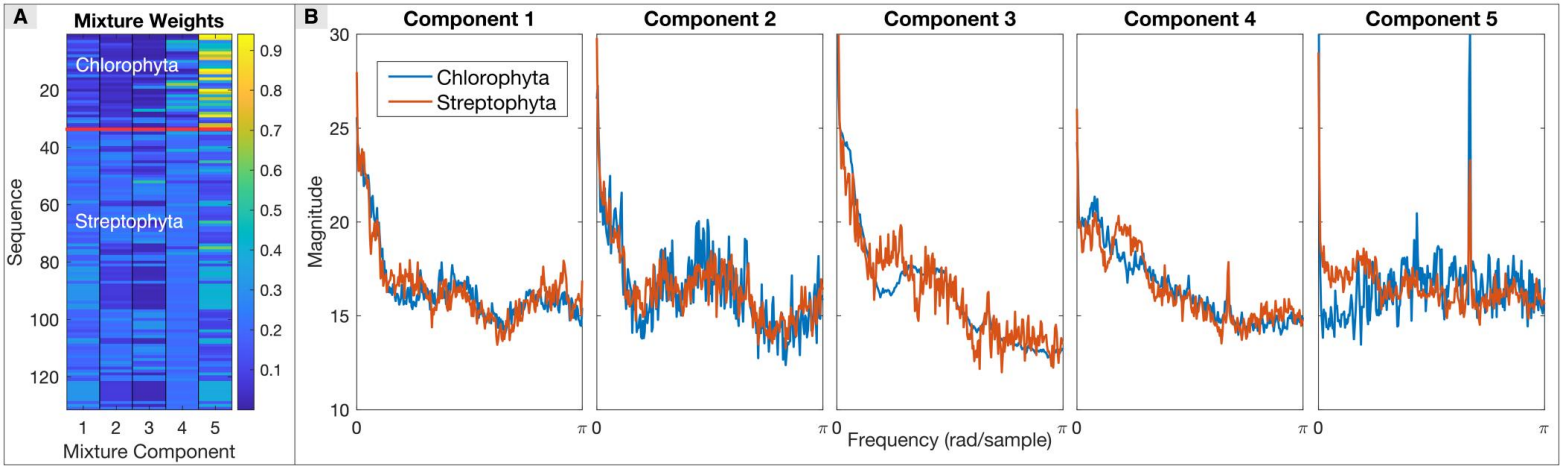
SpecGMM’s application to classify plant species: A five-mixture component GMM was built using 256-dimensional magnitude spectra (0 to *π* rad/sample) from DNA sequences of two plant species, Chlorophyta and Streptophyta. (A) The figure displays a heatmap of mixture weights for training sequences from the two plant species represented in a matrix of sequences (rows) against five mixture components (columns). (B) The figure represents the mixture component means of adapted Gaussian Mixture Models for selected sequences from Chlorophyta and Streptophyta, visually demonstrating how the means of the mixture components differ between the two species.

To select SpecGMM’s hyperparameters, we took the window size recommendation from an earlier GSP study (Vaidyanathan 2004) and combined it with the optimal number of mixture components learnt from an mtDNA dataset having two plant categories (see Supplementary Methods: Section 1.6). These plant categories—Chlorophyta and Streptophyta—comprised sequences of highly varying lengths. We found that the window size of 351, a window shift of 99 nucleotides, an FFT order of 512, and a 5-component UBM-GMM performed reasonably well (see Supplementary Table S2) and displayed strong discriminative capabilities (see Fig. 2). Notable differences were observed in the mixture component weights during MAP adaptation for training sequences from the two categories, particularly for components 4 and 5 (see Fig. 2A). Furthermore, visual differences between the plant categories were evident in the means of the adapted GMMs, especially that of mixture components 3–5 (see Fig. 2B). These results show that SpecGMM’s fixed-dimensional representation can be used for classification in settings where sequences are of highly varying lengths. The hyperparameters underlying this representation were applied as is for analyzing other sequence datasets too.

### 3.2 SpecGMM’s comparative performance and robustness to various factors

SpecGMM’s performance was benchmarked against a baseline that utilized PP representation, median length normalization, and PCC-based distance measures (Randhawa *et al.* 2019). Across benchmark datasets from three different studies, SpecGMM was found to perform better than or comparable to the baseline (see Table 1). SpecGMM achieved improvements in average classification accuracies of up to 18.88% for the LD classifier and 23.15% for the LSVM classifier, compared to the baseline on test sequences. Additionally, the performance of SpecGMM was not compromised by sequence length variability, as indicated by the Median Absolute Deviation (MAD), especially in the case of BacteriaTest dataset, reconfirming SpecGMM’s capability to handle sequences of varying lengths effectively. Detailed classification results, including metrics like standard deviation, precision, recall, specificity, and F1-score, are provided in Supplementary File D3. A comparative analysis of various numerical representations across different datasets is provided in Supplementary File D4, which confirms PP representation to be the best-performing one.

**Table 1.** Comparative classification accuracies using baseline and SpecGMM methods for benchmark datasets: Average classification accuracies (in %) for independent test sets across four folds for Linear Discriminant (LD) and Linear SVM (LSVM) classifiers are reported for various benchmark datasets.

Dataset	#Cls	Chance Acc.	Baseline Acc.		SpecGMM Acc.		delta		Med. of Seq. Len.	MAD
			LD	LSVM	LD	LSVM	LD	LSVM		
**Datasets with different properties (** Randhawa *et al.* 2019 **)**										
Primates	2	65.35	95.95	98.63	97.3	100	1.35	1.37	16554	50
Protists	3	37.63	91.13	74.35	96.85	97.5	5.72	**23.15**	35660	8000
Fungi	3	39.49	78.15	73.2	93.78	95.13	**15.63**	**21.93**	39154	13337
Plants*	2	62.21	91.35	86.18	91.95	93.08	0.6	6.9	128211	96761
Amphibians	3	42.93	87.95	90.05	97.25	100	9.3	9.95	17271	848
Insects	7	19.07	82.5	85.3	93.78	99	**11.28**	**13.7**	15529	302
threeClasses	3	39.5	86.68	99.55	99.63	99.95	**12.95**	0.4	16361	489
Vertebrates	5	35.27	85	97.85	98	100	**13**	2.15	16616	135
BacteriaTest (bacterial genome)	3	51.16	76.45	92.8	92.68	98.53	**16.23**	5.73	70992	37385
Birds_Fish_Mammals	3	45.9	98.13	99.98	99.98	100	1.85	0.02	16606	111
Dengue (viral genome)	4	31.4	97.18	99.88	100	100	2.82	0.12	10676	31
Mammalia	8	18.42	78.93	90.38	96.6	99.38	**17.67**	9	16537	161
**Datasets at different taxonomy levels (** Randhawa et al., 2019 **)**										
Domain-Kingdom (Eukaryota)	4	82.3	81.63	95.45	91.5	98.83	9.87	3.38	16580	409
Domain-Kingdom (Eukaryota_noProtists)	3	86.35	88.5	97.23	93.5	99.08	5	1.85	16573	373
Kingdom-Phylum (Animalia)	7	48.81	74.95	94.73	93.83	98.75	**18.88**	4.02	16553	292
Phylum-Subphylum (Chordata)	3	98.5	96.38	99.55	99.93	99.88	3.55	0.33	16615	137
Subphylum-Class (Vertebrata)	5	35.27	85	97.85	98	100	**13**	2.15	16616	135
Class-Subclass (Actinopterygii)	3	96.82	98.95	99.8	100	99.95	1.05	0.15	16589	69
Subclass-Superorder (Neopterygii)	7	37.26	81.3	93.23	95.88	97.73	**14.58**	4.5	16597	65
Superorder-Order (Ostariophysi)	3	69.82	86.15	98.88	99.75	100	**13.6**	1.12	16597	24
Order-Family (Cypriniformes)	5	63.86	89.45	96.58	99.85	100	**10.4**	3.42	16601	20
Family-Genus (Cyprinidae)	6	18.09	89.33	80.8	95.35	96.5	6.02	**15.7**	16597	10
Subfamily-Genus (Acheilognathinae)	2	50.09	100	100	100	100	0	0	16600	12
**Viral sequence datasets at different taxonomy levels (** Randhawa et al., 2020 **)**										
Test-1 (11 viral families + Riboviria)	12	10.88	71.73	86.45	82.7	90.98	**10.97**	4.53	7350	4559
Test-2 (families of realm Riboviria)	12	10.74	60.63	79.63	78.55	83.58	**17.92**	3.95	7486	2489
Test-3a (genera of family Coronaviridae)	4	44.14	90.78	87.9	99.03	98.1	8.25	**10.2**	29704	1178
Test-3b (genera of family Coronaviridae)	3	48.04	91.38	91.88	99	99	7.62	7.12	29704	860
Test-4 (subgenera of Betacoronavirus)	4	32.66	97.43	91.83	100	100	2.57	8.17	30161	559
Test-5 (Test-4 + SARS-CoV-2)	5	25.03	96.65	95.35	98.75	100	2.1	4.65	29891	280
Test-6 (Sarbecovirus + SARS-CoV-2)	2	52.57	97.35	97.35	97.23	97.23	−0.12	−0.12	29749	76

Accuracies are compared against a random classifier’s expected accuracy, calculated based on class proportions. The table also includes the median of sequence lengths and their Median Absolute Deviation (MAD) to indicate variability within each dataset. All sequences were processed using the PP representation. The baseline method used median length normalization and PCC-based distance measures, while SpecGMM utilized fixed hyperparameters: a window size of 351, window shift of 99, FFT order of 512, and a 5-component UBM-GMM. The delta column quantifies the performance difference between SpecGMM and the baseline for both classifiers. The delta values that are at least 10 are marked in bold font. The average delta values for LD and LSVM, excluding the Plants dataset used for hyperparameter tuning and including results from 16S rRNA datasets in Table 2, were 9.45% and 4.81%, respectively.

We also performed a few additional analyses to test the robustness of the observed performance trends. SpecGMM was run with four other classifiers (besides LD and LSVM) used in the baseline study; and across these six classifiers, SpecGMM continued to perform better than baseline in 29–35 of the total 36 benchmark datasets (see Supplementary File D3). To address potential information leakage issues arising from high similarity between training vs. testing set sequences, we performed homology reduction using GraphPart (Teufel *et al.* 2023), and evaluated SpecGMM vs. baseline on the homology-reduced datasets. As shown in Supplementary File D3, the better performance of SpecGMM over the baseline also prevailed after homology reduction.

### 3.3 SpecGMM aids classification of 16S rRNA sequences

We applied SpecGMM to classify 16S rRNA sequences across different taxonomic levels, using the same hyperparameters optimized for the benchmark datasets. As outlined in Table 2, SpecGMM consistently outperformed the baseline method at all taxonomy levels, except at the Species level for the LSVM classifier. At this level, SpecGMM’s LD classifier surpassed the baseline by 13.27%, while the baseline LSVM classifier performed slightly better. For all 16S rRNA datasets combined, SpecGMM achieved an accuracy improvement of up to 35.55% and 4.28% for LD and LSVM classifiers, respectively.

**Table 2.** Comparative classification accuracies using baseline and SpecGMM methods for 16S datasets: The fields are same as in Table 1.

Dataset	#Classes	Chance Acc.	Baseline Acc.		SpecGMM Acc.				Median of Seq. Lengths	MAD
			LD	LSVM	LD	LSVM	LD	LSVM		
					WinLen = 351 WinShift = 99		WinLen = 63 WinShift = 9			
Phylum (Kingdom: Bacteria)	12	8.72	74.18	85.13	**84.23**	**88.83**	82.98	87.8	1473	31
Class (Phylum: Firmicutes)	3	5.8	89.9	95.88	93.88	**97.05**	**94.15**	96.95	1474	60
Order (Class: Bacilli)	5	1.95	77.73	92.45	**93.15**	**94.75**	91.2	94.7	1512	44
Family (Order: Bacillales)	4	1.39	60.58	94.35	**96.13**	**98.63**	95.05	98.08	1478	41
Genus (Family: Bacillaceae)	3	1.5	92.08	97.53	97.53	**99.85**	**98.73**	99.55	1469	36
Species (Genus: Bacillus)	9	2.46	36.58	63.98	49.85	57.83	**62.75**	**67.15**	1537	19

Accuracies for two different window sizes (WinLen) and shifts (WinShift) are reported for SpecGMM method. Maximum average accuracies for each classifier are marked in bold font. Detailed results are available in Supplementary File D3.

Recognizing the importance of HVRs in 16S rRNA sequences for species classification (Yang *et al.* 2016), we adopted a shorter window size and shift than the default 351 and 99 nucleotides to enhance resolution across sequence length (see Supplementary Methods: Section 1.6). For species-level classification, the accuracy of SpecGMM with a window size of 63 nucleotides, a shift of 9, and an FFT order of 64 was better than that of the GSP baseline by 26.17% and 3.17% for LD and LSVM classifiers, respectively.

### 3.4 SpecGMM framework reveals discriminative HVRs of 16S rRNA

**Figure 3.**
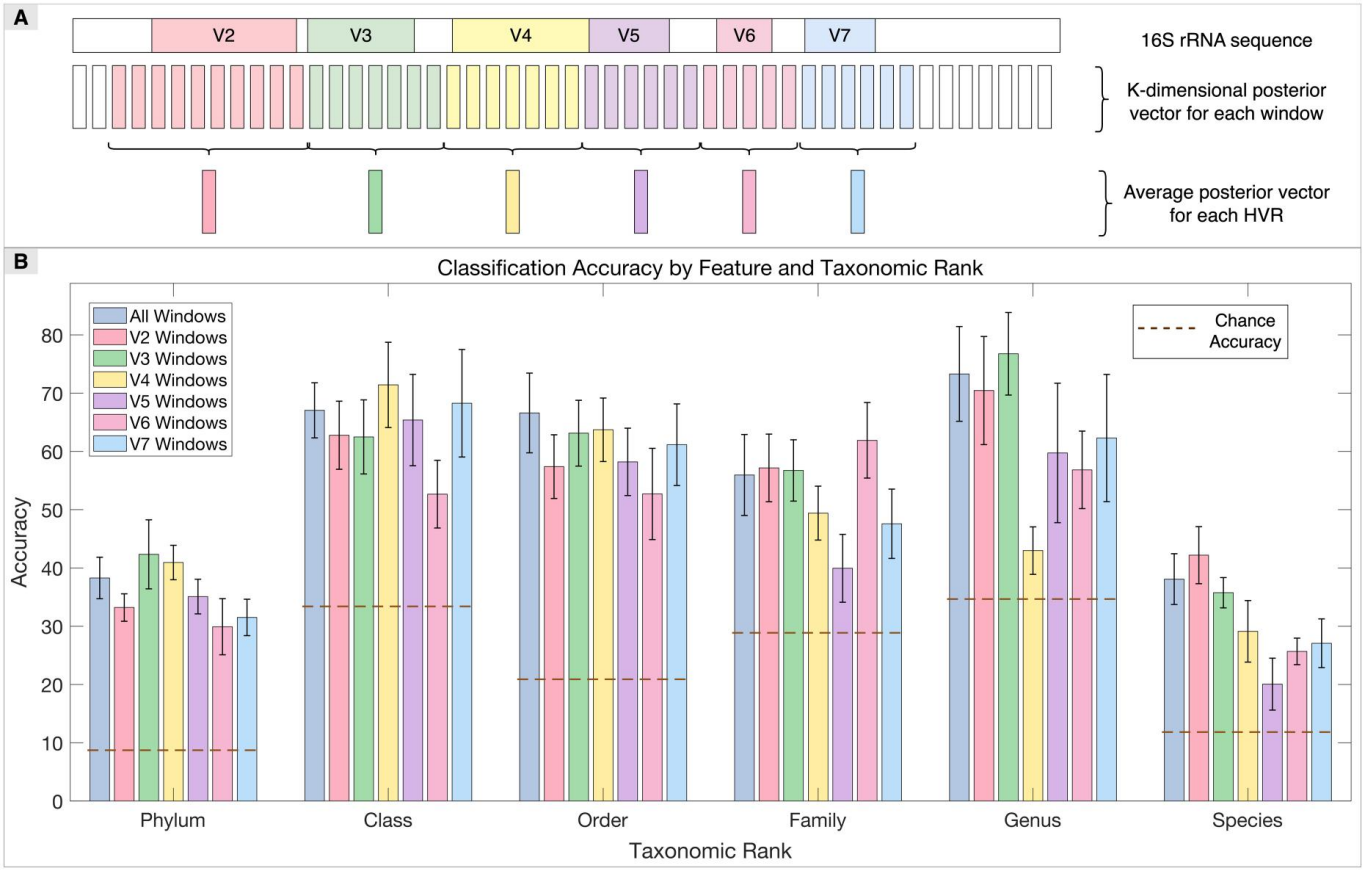
Discriminative hypervariable regions (HVRs) in 16S rRNA sequences according to SpecGMM for taxonomic classification: (A) For each sequence, windows are assigned to each HVR (V2–V7) based on maximum overlap criterion. Posterior probabilities are computed for each window (using the magnitude spectrum of the window as detailed in Supplementary Algorithm 2). This generates a K-dimensional posterior vector per window, with respect to the K mixture-component UBM-GMM. The posterior vectors of the windows assigned to an HVR can then be averaged to get a posterior representation of the HVR. These average posteriors are used as features for classification. (B) The figure compares taxonomic classification accuracies using HVR-based features. The brown dotted lines show the chance accuracies.

To further investigate SpecGMM’s discriminative capabilities and interpretability, we examined the impact of different regions of the 16S rRNA sequences on classification accuracy, as a post-hoc analysis. Specifically, we considered 16S rRNA HVRs and assigned each sequence window to an HVR based on the maximum overlap criterion. We derived average posterior representations from window-based posteriors during the MAP adaptation process, as depicted in Fig. 3A. These K-dimensional posterior vector representations from a K-component GMM served as features for classifying different taxonomic levels.

The rationale for choosing this posterior representation is driven by the purpose of the current analysis, which is to assess the discriminatory power of individual HVR regions using the SpecGMM framework. Another possible representation would be to derive a mean supervector per HVR, but this would not be effective due to the insufficient number of windows per HVR.

As illustrated in Fig. 3B, classification accuracies varied significantly across HVRs at different taxonomic levels/ranks. V3 and V4 showed particularly high discriminative power at most ranks, especially at higher ones, consistent with prior studies (e.g., Yang *et al.* 2016). At the Species and Genus levels, V2 and V3 were notably informative, aligning with findings by Bukin *et al.* (2019). In some cases, V5–V7 also demonstrated considerable discriminative ability. While using features from the entire sequence yielded robust results, each HVR individually exceeded chance accuracy, highlighting their value in taxonomic classification. However, accuracies decreased at more specific taxonomic levels, like species classification, emphasizing the challenge of distinguishing closely related organisms.

### 3.5 Spectrogams help interpret SpecGMM results and visualize signatures

**Figure 4.**
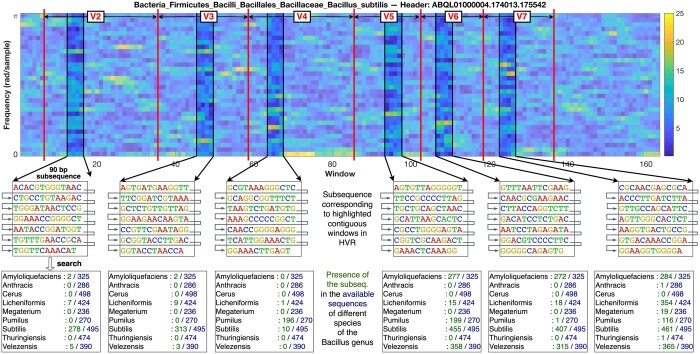
Spectrogram analysis of a representative *Bacillus subtilis* 16S rRNA sequence: A spectrogram shows the magnitude spectra obtained from windows along the length of a given sequence as columns of a heatmap. For the sequence here, WinLen = 63 and WinShift = 9 yield 160+ windows (with each window’s magnitude spectrum shown as a heatmap column) and 32 frequency components (heatmap rows). For each HVR (see Fig. 3A), we choose four contiguous windows with some prominent frequency components, and extract the DNA substring (contiguous subsequence) spanned by these windows. We search this DNA substring across sequences belonging to genus *Bacillus*, and report the number of matching sequences from different species of the *Bacillus* genus. Note that the DNA substring is 90-nucleotide long and hence shown inside a box in a wrap-around form; the corresponding four consecutive windows are also highlighted in the spectrogram above the box. Refer to Supplementary Videos SV1–SV9 for visualization of common spectrogram patterns across sequences of given species.

Spectrogram analysis, which visualizes the frequency versus position characteristics of DNA sequences, facilitates the identification of local spectral patterns. In our study, spectrograms were generated for 16S rRNA sequences from various species within the genus *Bacillus*. Figure 4 depicts spectrogram for a representative sequence of the species *Bacillus subtilis*. Further, videos were created using the spectrograms of 100 selected sequences of each available species of genus *Bacillus* (see Supplementary Videos SV1–SV9). These spectrogram videos aided visualization of species-specific signatures. Notably, subsequences from the V2 and V3 regions were predominantly found in *Bacillus subtilis* sequences. For instance, the subsequence corresponding to the V2 region appeared in 278 out of 495 available *Bacillus subtilis* sequences, but was less prevalent or absent in available sequences from other species within the genus. A similar pattern was observed for the V3 region. In contrast, subsequences from the V4–V7 regions were also present in sequences from other species. These findings align with our previous HVR analysis (refer to Fig. 3B), which highlighted the discriminative potential of the V2 and V3 regions in distinguishing species within the genus *Bacillus*.

The results taken together show that our SpecGMM approach not only demonstrates superior classification accuracy compared to the baseline but also enriches our understanding of the variations in spectral characteristics over the sequence length. The integration of spectral/spectrogram analysis into our methodology provides a powerful tool for visually interpreting complex DNA patterns, thereby enhancing the interpretability of our results.

## 4 Discussion

Our study introduces SpecGMM, a novel approach that addresses the challenges associated with spectral representation of variable-length DNA sequences in the context of taxonomic classification. Through the integration of signal processing techniques with GMM, SpecGMM not only enhances the discriminative power of spectral features for genomic sequences but also suggests that the sequences possess species-specific signatures.

Our comprehensive evaluation across diverse datasets, including mitochondrial DNA, bacterial and viral genomes, and particularly 16S rRNA sequences, showed that SpecGMM performs better than or comparable to the baseline GSP method. The hyperparameters optimized using the Plants (mtDNA) dataset were used as is for other datasets having various sequence types, yet SpecGMM performed consistently across these datasets, showcasing its robustness. Moreover, the interpretability offered by SpecGMM, especially in analyzing 16S rRNA sequences and visualizing their local spectral patterns using spectrogram videos, provided insights into the discriminative power of different 16S rRNA HVRs at different taxonomic ranks.

There are many paradigms to taxonomic classification, such as ones based on sequence or k-mer alignment, deep learning, or GSP-guided machine learning proposed in this work. A fair comparison of methods from different paradigms is challenging—for instance, k-mer approaches like Kraken 2 (Wood *et al.* 2019) rely on a reference database, and their accuracy depends on how well the reference database captures the species in the benchmark dataset (Lu *et al.* 2022); whereas our SpecGMM follows a training-testing machine learning paradigm. Nevertheless, to place SpecGMM in the context of other popular tools, we have compared it to the k-mer based method Kraken 2 and a deep learning based method, DNABERT-S (Zhou *et al.* 2024), and discuss these results next:

- SpecGMM is comparable to Kraken 2 on genus-level classification and outperforms Kraken 2 on species-level classification of the 16S rRNA benchmark datasets (Supplementary Table S3). This observation aligns with Kraken 2’s known limitation of misclassifying sequences at the species level, when sister species of the same genus are present in the reference database (Wood *et al.* 2019).
- In the same 16S rRNA bacterial benchmarks, we compared SpecGMM, which represents each sequence by a supervector from a UBM-GMM model, to DNABERT-S, which represents each sequence by an embedding from a species-aware deep learning model trained on microbial sequences (bacteria, fungi, and viruses). SpecGMM was significantly faster in generating sequence representations; and performed comparable to or better than DNABERT-S in all datasets except for one dataset at species level (Supplementary Table S4), where DNABERT-S outperformed SpecGMM likely due to its specialized learning for species-level classification. Additionally, when tested on eukaryotic datasets with sequences from species not seen when learning the representation models (UBM-GMM or DNABERT-S’ model), SpecGMM consistently outperformed DNABERT-S (Supplementary Table S5). These findings show the generalizability of SpecGMM to unseen data. However, direct comparisons are challenging because the models differ in learning strategies and training data, and DNABERT-S was trained specifically for species-level classification tasks (see captions of Supplementary Tables S4 and S5 for details).

SpecGMM’s primary distinction from other paradigms discussed above lies in its efficient integration of GSP with GMMs. SpecGMM ensures robust handling of variable-length sequences without the need for padding or truncation. Moreover, it provides unique interpretability of the spectral features of DNA sequences, as can be seen from our 16S rRNA analysis, offering clearer insights into the features used for classification compared to deep learning models, which often lack transparency and require high computational resources.

While our method has shown performance improvement on several benchmark datasets, handling the computational demands of large datasets remains challenging. Nevertheless, the promising results achieved with SpecGMM encourage its application and potential effectiveness in broader genomic studies that are yet to be explored. Future research will focus on further enhancing SpecGMM’s efficiency and scalability on even more diverse sets of sequences not well studied in the literature, and on understanding any classification biases of SpecGMM. Regarding the latter, we did not find any significant sequence length bias affecting the classification of an analyzed 16S rRNA dataset. To derive optimal sequence lengths for classification, future work can focus on a more detailed analysis of a wider range of datasets. Another promising avenue is the exploration of more nuanced spectral features within genomic sequences, using advanced signal processing techniques, highlighting discriminative DNA regions and studying their biological significance. To conclude, SpecGMM represents a promising step forward in the field of signal processing-based genomic classification, offering both improved accuracy and deeper insights into the spectral characteristics of DNA sequences.

## Supplementary Material

vbae171_Supplementary_Data
