# Supplementary material for: SpecGMM: Integrating Spectral analysis and Gaussian Mixture Models for taxonomic classification and identification of discriminative DNA regions
Source: Bioinform Adv. 2024 Nov 5;4(1):vbae171. doi: 10.1093/bioadv/vbae171 (PMC11631429; doi:10.1093/bioadv/vbae171)
Supplement: vbae171_Supplementary_Data [file vbae171_supplementary_data.pdf]

# Supplementary Material
## for "SpecGMM: Integrating Spectral analysis and Gaussian Mixture Models for taxonomic classification and identification of discriminative DNA regions"


1 Supplementary Methods 1
1.1 Background on Discrete Fourier Transform (DFT) 1
1.2 Magnitude spectrum analysis of genomic signals with PP representation 1
1.3 Algorithm 1: Expectation-Maximization (EM) for Gaussian Mixture Model (GMM) 2
1.4 Algorithm 2: Maximum a Posteriori (MAP) Adaptation for Universal Background Model - Gaussian Mixture Model (UBM-GMM) 2
1.5 Time-Complexity analysis of SpecGMM method 2
1.6 Hyperparameter selection 3
1.7 Preprocessing of 16S rRNA dataset 3

2 Supplementary Data Files 4

3 Supplementary Figures 5

4 Supplementary Tables 6

5 Supplementary Videos 8


## Supplementary Methods

### Background on Discrete Fourier Transform (DFT)

The Discrete Fourier Transform (DFT) is a fundamental operation in signal processing. It transforms the time domain sequence information into its frequecy domain counterpart. It is defined for a sequence $x[n]$ of length $N$ as follows:

$$X[k] = \sum_{n=0}^{N-1} x[n] \cdot e^{-\frac{j2\pi}{N}kn}, \quad k = 0, 1, \ldots, N-1 \tag{1}$$

$$e^{-\frac{j2\pi}{N}kn} = \cos\left(\frac{2\pi}{N}kn\right) - j\sin\left(\frac{2\pi}{N}kn\right) \tag{2}$$

where

- $X[k]$ is the $k$-th frequency component,
- $x[n]$ is the $n$-th the time domain sample,
- $N$ is the total number of samples, and
- $j$ is the imaginary unit with $j^2 = -1$.

The Fast Fourier Transform (FFT) algorithm can greatly speed up the computation of the DFT, especially when the sequence length is a power of two. The sequence can be padded with zeros, if $N$ is not a power of two, to increase its length to the next power of two to improve FFT's efficiency.

### Magnitude spectrum analysis of genomic signals with PP representation

For a given genomic sequence, we employed Purine-Pyrimidine (PP) numerical representation, where the nucleotides are converted to numerical values as follows:

$$\text{Adenine (A) and Guanine (G)} \rightarrow -1 \text{ (Purines)},$$

$$\text{Thymine (T) and Cytosine (C)} \rightarrow +1 \text{ (Pyrimidines)}.$$

This conversion of sequence to signal facilitates the application of DFT, as shown in Equation 1. In this case, $x[n]$ represents the $n$-th nucleotide in the PP representation, and $N$ is the total number of nucleotides. Let $F$ denote the FFT order, which is set to the closest power of two that is greater than or equal to $N$. This choice ensures that the FFT is computed efficiently.

The magnitude spectrum, reflecting the frequency components' strengths within the genomic signal, is obtained by determining the magnitude of each Fourier coefficient:

$$M[k] = |X[k]| = \sqrt{\text{Re}(X[k])^2 + \text{Im}(X[k])^2}, \quad k = 0, 1, \ldots, F-1. \tag{3}$$

The Fourier Transform of a real-valued signal, such as our PP representation of genomic sequences, exhibits symmetry: $X[k] = X[F-k]^*$ for $k = 1, 2, \ldots, F-1$. Here, $^*$ denotes the complex conjugate. This symmetry implies that the magnitude spectrum is also symmetric, with $M[k] = M[F-k]$. Therefore, only the first half of the spectrum (up to $F/2$ coefficients) is needed to fully represent the signal. This is particularly important in reducing computational load and data redundancy.

For example, if $N < 512$ is not a power of two and $F$ is set to 512 to ensure that the sequence length is compatible with the FFT algorithm, analyzing only the first 256 coefficients (from 0 to $\pi$ radians per sample) suffices. This subset fully encapsulates the signal's frequency characteristics due to the aforementioned symmetry.

## Algorithm 1: Expectation-Maximization (EM) for Gaussian Mixture Model (GMM)

**Algorithm 1** Expectation-Maximization for GMM

1: Input: Number of mixtures – $K$, Window length $W$, Dimension of each magnitude spectrum $D = 2^{\lceil \log_2(W) \rceil - 1}$, Data (Magnitude spectra over $n$ sliding windows) – $X = \{m_1, m_2, \ldots, m_n\}$ with $m_i \in \mathbb{R}^D$,
2: Output: K-component GMM with parameters $(\pi_k, \mu_k, \Sigma_k)$ where $\mu_k \in \mathbb{R}^D$, $\Sigma_k \in \mathbb{R}^{D \times D}$, and $\pi_k \in \mathbb{R}$
3: Get initial mixture component parameters by running K-means for a few iterations
4: **E-step:** Calculate the responsibilities (posteriors) and effective number of points in mixture components
5: $\gamma_{nk} \leftarrow \frac{\pi_k \mathcal{N}(m_n|\mu_k, \Sigma_k)}{\sum_{j=1}^{K} \pi_j \mathcal{N}(m_n|\mu_j, \Sigma_j)}$ ▷ $\gamma_{nk}$ is the responsibility of n-th point towards k-th mixture component
6: $N_k \leftarrow \sum_{n=1}^{N} \gamma_{nk}$ ▷ $N_k$ is the effective number of points in that particular mixture component
7: **M-step:** Estimate the means and covariance matrices for each mixture component
8: $\pi_k \leftarrow \frac{N_k}{N}$
9: $\mu_k \leftarrow \frac{1}{N_k} \sum_{n=1}^{N} \gamma_{nk} m_n$
10: $\Sigma_k \leftarrow \frac{1}{N_k} \sum_{n=1}^{N} \gamma_{nk} (m_n - \mu_k)(m_n - \mu_k)^T$
11: Calculate joint log-likelihood using these estimated parameters
12: $ln\ p(X|\lambda) \leftarrow \sum_{n=1}^{N} \ln \left\{ \sum_{k=1}^{K} \pi_k \mathcal{N}(m_n|\mu_k, \Sigma_k) \right\}$ ▷ Calculation of joint log-likelihood
13: Repeat E-step and M-step until log-likelihood converges to a threshold

## Algorithm 2: Maximum a Posteriori (MAP) Adaptation for Universal Background Model - Gaussian Mixture Model (UBM-GMM)

**Algorithm 2** Maximum a Posteriori (MAP) Adaptation for UBM-GMM

1: Input: UBM-GMM parameters $\lambda_{UBM} = \{\mu_k^{ubm}, \pi_k^{ubm}, \Sigma_k^{ubm}\}$, data points $X = \{m_1, m_2, \ldots, m_n\}$, number of mixtures $K$, dimension of feature (data points from a sequence) $D$
2: Output: Mean-adapted GMM $\{\mu_k, \pi_k^{ubm}, \Sigma_k^{ubm}\}$ where $\mu_k \in \mathbb{R}^D$, $\pi_k^{ubm} \in \mathbb{R}$, $\Sigma_k^{ubm} \in \mathbb{R}^{D \times D}$ ▷ Only mean $\mu_k$ is adapted
3: $\gamma_{nk} \leftarrow \frac{\pi_k^{ubm} \mathcal{N}(m_n|\mu_k^{ubm}, \Sigma_k^{ubm})}{\sum_{j=1}^{K} \pi_j^{ubm} \mathcal{N}(m_n|\mu_j^{ubm}, \Sigma_j^{ubm})}$ ▷ Compute responsibilities (posteriors) for each data point
4: $N_k \leftarrow \sum_{n=1}^{N} \gamma_{nk}$ ▷ Effective number of data points in each mixture component
5: $\pi_k^{new} \leftarrow \frac{N_k}{N}$
6: $\mu_k^{new} \leftarrow \frac{1}{N_k} \sum_{n=1}^{N} \gamma_{nk} m_n$
7: $\alpha^P \leftarrow \frac{N_k}{N_k + \rho P}$ ▷ Combine new statistics with old using data-dependent mixing coefficient $\alpha^P$. $\rho = 16$ is the relevance factor in SpecGMM analysis
8: $\mu_k \leftarrow \alpha^P \mu_k^{new} + (1 - \alpha^P) \mu_k^{ubm}$ ▷ In our study, only mean adaptation is performed. Stack means $\mu_k$s to get a mean supervector of dimension KD for the sequence

## Time-Complexity analysis of SpecGMM method

In our SpecGMM approach, the computational complexity is derived from several key procedures. The first procedure involves sliding a window across DNA sequences to extract magnitude spectra. Given an average sequence length $L$, a window length $W$, a hop length $H$, and $S$ sequences, the time complexity for this step is $\mathcal{O}\left(S \cdot \left(\frac{L}{H}\right) \cdot W \cdot \log(W)\right)$.

The training of the UBM-GMM is the next step. With $N = S \cdot \left(\frac{L}{H}\right)$ training vectors, a feature dimensionality of $D = 2^{\lceil \log_2(W) \rceil - 1}$ considering angular frequencies in the range 0 to $\pi$ rad/sample in magnitude spectra, $K$ mixture components, and $I_{\text{UBM-GMM}}$ iterations for convergence, the time complexity associated with this phase is $\mathcal{O}(I_{\text{UBM-GMM}} \cdot N \cdot K \cdot D)$, assuming a diagonal covariance matrix.

For classification, the Linear Discriminant Analysis (LDA) entails a complexity of $\mathcal{O}(S \cdot (K \cdot D)^2 + (K \cdot D)^3)$, where $S$ denotes the number of sequences, and $K \cdot D$ represents the dimensionality of the feature vectors (mean supervectors).

Finally, the Support Vector Machine (SVM) classifier, with feature vectors of dimension $K \cdot D$ and $S$ sequences, results in a complexity of $\mathcal{O}(\max(S, K \cdot D) \cdot \min(S, K \cdot D)^2)$ (Chapelle, 2007), considering the worst-case scenario when all the sequence-specific feature vectors end up being support vectors.

## Hyperparameter selection

For our SpecGMM framework, the selection of hyperparameters such as window size, window shift, and the number of mixture components in UBM-GMM is crucial to balance computational efficiency with classification performance. We selected a window size of 351 nucleotides, based on empirical evidence from a prior study (Vaidyanathan, 2004), to capture local periodicity effectively in genomic sequences. Further, we set the FFT order to 512, the nearest power of two. We set the window shift to 99 nucleotides to ensure significant overlap between consecutive windows, which is crucial for maintaining continuity in spectral features. We employed a 5 mixture-component UBM-GMM with diagonal covariance, as this configuration provided an optimal balance between computational efficiency and classification accuracy. This determination was based on performance evaluations of configurations ranging from 2 to 10 mixture components, as detailed in Suppl. Table S2.

For analyses involving the 16S rRNA dataset, a shorter window size of 63 nucleotides was selected to adequately cover different hypervariable regions (HVRs), particularly accommodating the shortest HVR — V6 — which has an average length of 84 nucleotides (See Table S1). The window shift for this dataset was reduced to 9 to ensure sufficient coverage and overlap between consecutive windows, with an FFT order set to 64, the nearest power of two.

We used the same classifier-specific hyperparameters as used in the baseline method (Randhawa et al., 2019). These classifier settings, combined with the SpecGMM feature extraction settings, formed a robust methodology for classifying genomic sequences across diverse datasets.

## Preprocessing of 16S rRNA dataset

The 16S IT-GDB (Hsieh et al., 2022) dataset contained sequence with missing labels for certain taxonomic levels. Moreover, not all sequences contained all the hypervariable regions (HVR). The database's FASTA file containing 110,780 sequences and a CSV file containing the corresponding taxonomy labels – Kingdom to Species, were used for preprocessing. Our analysis focused on the Kingdom Bacteria. The QIIME2 toolkit (Bolyen et al., 2019) was used to obtain the HVRs V2 to V7 using the corresponding primers available from (Chaudhary et al., 2015) (Supporting Information: Table A). Sequences containing V2 to V7 HVRs were retained. We excluded the V1, V8, and V9 regions from our analysis due to inconsistencies in primer information across the literature. We selected 200 to 500 sequences for each taxonomic category, that contained all the targeted HVRs (V2 to V7) and the taxonomy labels from Kingdom to Species, to avoid severe class imbalance. The sequences with missing taxonomy labels were filtered out.

## Supplementary Data Files

All the supplementary data files are available at this link: `https://github.com/BIRDSgroup/SpecGMM/tree/main/Supplementary%20Data%20Files`.

### *Suppl. File D1: Dataset Details*

The file contains the following details of the datasets used in the study.

- Number and names of classification categories for each dataset
- Number of sequences per category
- Proportion of each category and the analytically computed chance accuracies using the proportions
- Descriptive statistics for lengths of sequences of each category across all the datasets — maximum, minimum, mean, mode, standard deviation, mean absolute deviation, and median absolute deviation.

### *Suppl. File D2: 16S-ITGDB Taxonomy and HVR Information*

The file contains the taxonomy information (Kingdom to Species labels) and the start and end positions of the 16S rRNA HVRs obtained using the QIIME2 analysis.

### *Suppl. File D3: Comprehensive Results from SpecGMM Evaluations*

This file contains detailed results from three types of SpecGMM vs. baseline evaluations that we performed in this study:

D3a Classification results for all the datasets analysed in the study for Linear Discriminant (LD), Linear SVM (LSVM), Quadratic SVM (QSVM), FineKNN, Subspace Discriminant, and Subspace KNN classifiers comparing baseline and SpecGMM methods. The following performance metrics, computed over four folds, are available in the file:

- average accuracy
- standard deviation
- average weighted precision
- average weighted recall
- average weighted specificity
- average weighted F1-score

The details on how to compute these performance metrics are provided in the MATLAB code available at `https://github.com/BIRDSgroup/SpecGMM`.

D3b Results for the baseline vs. SpecGMM methods after performing homology reduction on our datasets using the GraphPart algorithm (Teufel et al., 2023) for different threshold values and different numbers of partitions. This was done to tackle the potential issue of information leakage due to the high similarity between train and test sets. Please note that GraphPart could partition only some of the datasets for certain values of thresholds and the number of partitions. The LD and LSVM classifiers were used in the analysis.

### *Suppl. File D4: Comparative analysis of numerical representations*

We compared SpecGMM's performance on various datasets from the baseline study using different numerical representations.

# Supplementary Figures

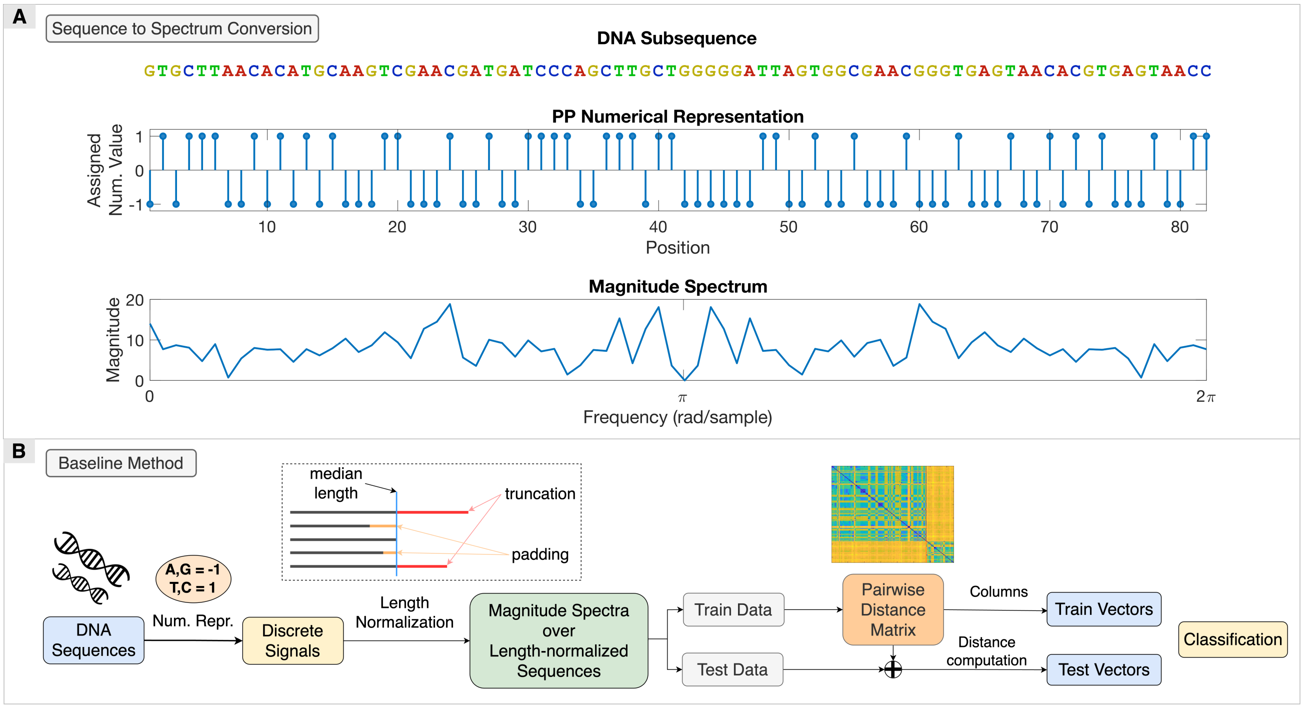


**Fig. S1. Background on GSP and baseline method:** A) PP representation is used to transform the DNA sequence into discrete signal as discussed in Section 2.1 in the main text. DFT is applied on the obtained signal to generate magnitude spectrum, which shows the strength of various frequency components within the signal. B) The figure depicts the baseline method (Randhawa et al., 2019), where DNA sequences of varying lengths are converted into discrete signals using the PP representation and are length-normalized by truncation/padding (see Section 2.1 in the main text). The length-normalized signals are then transformed into magnitude spectra. For classification, the dataset is divided into training and testing sets. A feature representation for the training data is created using a pairwise distance matrix, computed from the Pearson's correlation coefficient (PCC), using the formula (0.5-PCC/2), on the magnitude spectra representations. The same metric is applied to obtain feature representations for the test data, based on the distances between each test sequence and the train sequences. Various classifiers are then trained with the train features, and their performances are evaluated using the test data.

## Supplementary Tables

**Table S1. 16S rRNA QIIME2 analysis summary:** The number of 16S-ITGDB sequences in which QIIME2 recognized the given HVR is reported here. The details of the HVR primers used by QIIME2 are available from "Supporting Information: Table A" of Chaudhary et al. (2015).

| HVR | Number of sequences containing the HVR out of 110,780 available sequences | Average length of the HVRs identified in the sequences |
|---|---|---|
| V2 | 105,316 | 219.351 |
| V3 | 110,016 | 151.812 |
| V4 | 110,406 | 207.505 |
| V5 | 110,156 | 108.465 |
| V6 | 105,813 | 83.351 |
| V7 | 103,185 | 105.915 |

**Table S2. Selecting number of mixture components:** Different numbers of mixture components were evaluated on the Plants dataset from the baseline study — Randhawa et al. (2019). The window size was set to 351 and window shift was set to 99. As we increased the number of mixture components, the UBM-GMM training time increased. Moreover, the dimension of the mean supervectors also increases with the number of mixture components. Based on these constraints, we empirically set the number of mixture components to 5. This decision was informed by balancing computational efficiency and classification performance. The training time for 5 components provided a reasonable trade-off between time and accuracy, avoiding the significant increase in computational cost seen with 6 or more components, especially for larger datasets. Additionally, the 5-component model showed stable and high accuracy across different folds, minimizing the risk of overfitting. The experiments were run using MATLAB R2023a version on a Linux machine (x86 architecture) with 32 cores and around 450 GB RAM. MATLAB's tic and toc commands were used to measure running time of the codes.

| No. of UBM Mix. Comp. K | UBM Training Time (in sec) | | | | Average Accuracy and Std. Dev. (4-fold) | |
|---|---|---|---|---|---|---|
| | Fold-1 | Fold-2 | Fold-3 | Fold-4 | LD | LSVM |
| 2 | 7.82 | 15.66 | 9.07 | 10.47 | 91.95 (±2.66) | 92.5 (±3) |
| 3 | 28.55 | 33.63 | 21.4 | 57.52 | 91.375 (±2) | 93.08 (±2.86) |
| 4 | 41.27 | 124.99 | 29.02 | 65.71 | 92.5 (±4.75) | 93.07 (±3.29) |
| **5** | **65.01** | **80.82** | **55.62** | **64.98** | **91.95** (±2.66) | **93.08** (±2.86) |
| 6 | 84.19 | 144.26 | 66.75 | 77.66 | 92.525 (±2.58) | 94.8 (±3.42) |
| 7 | 83.81 | 115.91 | 93.79 | 88.72 | 93.65 (±4.14) | 94.25 (±1.15) |
| 8 | 106.99 | 129.79 | 88.43 | 103.59 | 93.65 (±4.14) | 94.25 (±1.15) |
| 9 | 111.98 | 118.92 | 121.88 | 164.56 | 93.075 (±3.29) | 91.95 (±2.66) |
| 10 | 141.4 | 163.45 | 184.04 | 171.09 | 91.325 (±4.5) | 95.4 (±3.65) |

**Table S3. Comparison of SpecGMM vs. Kraken 2 on 16S rRNA benchmark datasets:** Comparison of Kraken 2 with SpecGMM classifiers on genus and species-level classification accuracies using different 16S rRNA reference or training databases. As detailed in the manuscript, 16S-ITGDB (Hsieh et al., 2022) is an integrated database created using Greengenes (DeSantis et al., 2006), Silva (Quast et al., 2012), and RDP (Maidak et al., 1997) databases. Note that, when Kraken 2 was applied to all of our benchmark datasets, we found that, in some instances, Kraken 2 misclassified species not present in its reference database. To overcome this issue, which has also been reported before (Lu et al., 2022), and thereby enable a fairer comparison with SpecGMM, we focused only on benchmark datasets whose species were well-represented in Kraken 2's reference databases. This led us to prefer and focus on the 16S rRNA benchmark for Kraken 2 vs. SpecGMM comparison, over other benchmarks considered in this study.

| **Method** | **Accuracies at Genus and Species levels (in %)** | |
|---|---|---|
| | **Dataset** | |
| | **Genus (Family: Bacillaceae)** | **Species (Genus: Bacillus)** |
| Kraken 2 (with Greengenes as reference database) | 87.68 | 0 |
| Kraken 2 (with Silva as reference database) | 96.69 | 0 |
| Kraken 2 (with Kraken 2 Standard Database as reference database) | 69.36 | 19.69 |
| SpecGMM LD Classifier (with 16S-ITGDB as training database) | 98.73 | 62.75 |
| SpecGMM LSVM Classifier (with 16S-ITGDB as training database) | **99.55** | **67.15** |

**Table S4. Comparison of SpecGMM vs. DNABERT-S on 16S rRNA benchmark datasets.** Note that for each dataset (row in the table), SpecGMM's UBM-GMM is learnt using the training sequences in that dataset, whereas the DNABERT-S embedding model is pre-trained on a single dataset of microbial sequences (bacterial, viral, and fungal). "Sequence Representation Generation Time" reports the time taken to generate the sequence representation (supervector for SpecGMM or embedding for DNABERT-S) for each sequence in the training and test set, using the same computer workstation mentioned in Suppl. Table S2 caption. Delta values whose magnitude is above 5% are shown in bold-faced font.

| **Dataset** | **Accuracy (in %)** | | | | **Delta (SpecGMM – DNABERT-S)** | | **Sequence Representation Generation Time (seconds)** | |
|---|---|---|---|---|---|---|---|---|
| | **SpecGMM** | | **DNABERT-S** | | **LD** | **LSVM** | **SpecGMM** | **DNABERT-S** |
| | **LD** | **LSVM** | **LD** | **LSVM** | | | | |
| Phylum (Kingdom: Bacteria) | 82.98 | 87.8 | 67.95 | 87.33 | **15.03** | 0.47 | **21** | 1669 |
| Class (Phylum: Firmicutes) | 94.15 | 96.95 | 80.8 | 96.08 | **13.35** | 0.87 | **9** | 460 |
| Order (Class: Bacilli) | 91.2 | 94.7 | 87.03 | 94.05 | 4.17 | 0.65 | **18** | 1048 |
| Family (Order: Bacillales) | 95.05 | 98.08 | 87.1 | 98 | **7.95** | 0.08 | **10** | 475 |
| Genus (Family: Bacillaceae) | 98.73 | 99.55 | 98.95 | 99.93 | -0.22 | -0.38 | **11** | 438 |
| Species (Genus: Bacillus) | 62.75 | 67.15 | 69.55 | 77.33 | **-6.8** | **-10.18** | **22** | 1135 |

**Table S5. Comparison of SpecGMM vs. DNABERT-S on eukaryotic benchmark datasets.** Classification accuracies for benchmark datasets from Randhawa et al., 2019 are shown in the table. SpecGMM's UBM-GMM model in this table is learnt using a single 16S rRNA dataset (specifically the "Phylum (Kingdom: Bacteria)" dataset; unlike Suppl Table S4, where UBM-GMM was learnt separately for each benchmark dataset). DNABERT-S is pre-trained from microbial sequences as in Suppl. Table S4. Delta values whose magnitude is above 5% are shown in bold-faced font.

| **Eukaryotic datasets at different taxonomy levels (Randhawa et al., 2019)** | **Accuracy (in %)** | | | | **Delta (SpecGMM – DNABERT-S)** | |
|---|---|---|---|---|---|---|
| | **SpecGMM (UBM-GMM built on Bacterial 16S rRNA sequences)** | | **DNABERT-S (Model built on Bacterial, Fungi, and Viral sequences)** | | | |
| | **LD** | **LSVM** | **LD** | **LSVM** | **LD** | **LSVM** |
| KingdomToPhylum_Animalia | 91.68 | 97.5 | 87.08 | 96.88 | 4.6 | 0.62 |
| PhylumToSubphylum_Chordata | 99.85 | 99.73 | 99.825 | 99.9 | 0.025 | -0.17 |
| SubphylumToClass_Vertebrata | 95.88 | 99.78 | 83.375 | 98.55 | 12.505 | 1.23 |
| ClassToSubclass_Actinopterygii | 100 | 99.95 | 93.15 | 99.95 | **6.85** | 0 |
| SubclassToSuperorder_Neopterygii | 94.58 | 96.03 | 69.3 | 90.33 | **25.28** | **5.7** |
| SuperorderToOrder_Ostariophysi | 97.95 | 100 | 86.15 | 97.8 | **11.8** | 2.2 |
| OrderToFamily_Cypriniformes | 98.13 | 99.85 | 68.38 | 94.68 | **29.75** | **5.17** |
| FamilyToGenus_Cyprinidae | 92.98 | 94.1 | 91.83 | 87.75 | 1.15 | **6.35** |
| SubfamilyToGenus_Acheilognathinae | 100 | 100 | 100 | 100 | 0 | 0 |

## Supplementary Videos

### Spectrogram Videos for different species:

Videos created using spectrograms derived from 100 representative sequences of each species in the *Bacillus* genus dataset are available at this link — `https://bit.ly/SpecGMM-Spectrogram-Videos` and are listed below. The videos compare and contrast spectral pattern across sequences of the same species.

- Suppl. Video SV1: *Bacillus amyloliquefaciens*
- Suppl. Video SV2: *Bacillus anthracis*
- Suppl. Video SV3: *Bacillus cereus*
- Suppl. Video SV4: *Bacillus licheniformis*
- Suppl. Video SV5: *Bacillus megaterium*
- Suppl. Video SV6: *Bacillus pumilus*
- Suppl. Video SV7: *Bacillus subtilis*
- Suppl. Video SV8: *Bacillus thuringiensis*
- Suppl. Video SV9: *Bacillus velezensis*

## References

Bolyen, E., Rideout, J. R., Dillon, M. R., Bokulich, N. A., Abnet, C. C., Al-Ghalith, G. A., Alexander, H., Alm, E. J., Arumugam, M., Asnicar, F., et al. Reproducible, interactive, scalable and extensible microbiome data science using qiime 2. *Nature Biotechnology*, 37(8):852–857, 2019.

Chapelle, O. Training a support vector machine in the primal. *Neural Computation*, 19(5):1155–1178, 2007.

Chaudhary, N., Sharma, A. K., Agarwal, P., Gupta, A., and Sharma, V. K. 16S classifier: a tool for fast and accurate taxonomic classification of 16S rRNA hypervariable regions in metagenomic datasets. *PLOS ONE*, 10(2):e0116106, 2015.

DeSantis, T. Z., Hugenholtz, P., Larsen, N., Rojas, M., Brodie, E. L., Keller, K., Huber, T., Dalevi, D., Hu, P., and Andersen, G. L. Greengenes, a chimera-checked 16S rRNA gene database and workbench compatible with arb. *Applied and Environmental Microbiology*, 72(7):5069–5072, 2006.

Hsieh, Y.-P., Hung, Y.-M., Tsai, M.-H., Lai, L.-C., and Chuang, E. Y. 16S-ITGDB: An integrated database for improving species classification of prokaryotic 16S ribosomal RNA sequences. *Frontiers in Bioinformatics*, 2:905489, 2022.

Lu, J., Rincon, N., Wood, D. E., Breitwieser, F. P., Pockrandt, C., Langmead, B., Salzberg, S. L., and Steinegger, M. Metagenome analysis using the kraken software suite. *Nature Protocols*, 17(12):2815–2839, 2022.

Maidak, B. L., Olsen, G. J., Larsen, N., Overbeek, R., McCaughey, M. J., and Woese, C. R. The rdp (ribosomal database project). *Nucleic acids research*, 25(1):109–110, 1997.

Quast, C., Pruesse, E., Yilmaz, P., Gerken, J., Schweer, T., Yarza, P., Peplies, J., and Glöckner, F. O. The silva ribosomal rna gene database project: improved data processing and web-based tools. *Nucleic Acids Research*, 41(D1):D590–D596, 2012.

Randhawa, G. S., Hill, K. A., and Kari, L. ML-DSP: Machine Learning with Digital Signal Processing for ultrafast, accurate, and scalable genome classification at all taxonomic levels. *BMC Genomics*, 20(1):1–21, 2019.

Teufel, F., Gíslason, M. H., Almagro Armenteros, J. J., Johansen, A. R., Winther, O., and Nielsen, H. Graphpart: homology partitioning for biological sequence analysis. *NAR genomics and bioinformatics*, 5(4):lqad088, 2023.

Vaidyanathan, P. Genomics and proteomics: a signal processor's tour. *IEEE Circuits and Systems Magazine*, 4(4):6–29, 2004.
